# Mitigation Approaches to Combat the Flu Pandemic

**DOI:** 10.4103/0974-777X.56258

**Published:** 2009

**Authors:** Raman Chawla, Rakesh Kumar Sharma, Deepali Madaan, Neha Dubey, Rajesh Arora, Rajeev Goel, Shefali Singh, Vinod Kaushik, Pankaj Kumar Singh, Vivek Chabbra, Janak Raj Bhardwaj

**Affiliations:** *Division of CBRN Defense, Institute of Nuclear Medicine and Allied Sciences, Brig SK Mazumdar Marg, Delhi - 110 054, India*; 1 *High Throughput Screening Lab, Jubilant Biosys Ltd, “Jubilant Discovery Center”, #92, Industrial Suburb, 2^nd^ Stage, Industrial Area, Yeshwantpur, Bangalore - 560 022, India*; 2 *National Disaster Management Authority, NDMA Bhawan, A-1, Safdarjung Enclave, Delhi - 110 029, India*

**Keywords:** Anti-viral therapies, Flu, H1N1, H5N1, Pandemics, Vaccination

## Abstract

Management of flu pandemic is a perpetual challenge for the medical fraternity since time immemorial. Animal to human transmission has been observed thrice in the last century within an average range of 11-39 years of antigenic recycling. The recent outbreak of influenza A (H1N1, also termed as swine flu), first reported in Mexico on April 26, 2009, occurred in the forty first year since last reported flu pandemic (July 1968). Within less than 50 days, it has assumed pandemic proportions (phase VI) affecting over 76 countries with 163 deaths/35,928 cases (as on 15^th^ June 2009). It indicated the re-emergence of genetically reassorted virus having strains endemic to humans, swine and avian (H5N1). The World Health Organisation (WHO) member states have already pulled up their socks and geared up to combat such criticalities. Earlier outbreaks of avian flu (H5N1) in different countries led WHO to develop pandemic preparedness strategies with national/regional plans on pandemic preparedness. Numerous factors related to climatic conditions, socio-economic strata, governance and sharing of information/logistics at all levels have been considered critical indicators in monitoring the dynamics of escalation towards a pandemic situation.

The National Disaster Management Authority (NDMA), Government of India, with the active cooperation of UN agencies and other stakeholders/experts has formulated a concept paper on role of nonhealth service providers during pandemics in April 2008 and released national guidelines - management of biological disasters in July 2008. These guidelines enumerate that the success of medical management endeavors like pharmaceutical (anti-viral Oseltamivir and Zanamivir therapies), nonpharmaceutical interventions and vaccination development etc., largely depends on level of resistance offered by mutagenic viral strain and rationale use of pharmaco therapeutic interventions. This article describes the mitigation approach to combat flu pandemic with its effective implementation at national, state and local levels.

## INTRODUCTION

Human civilization is exposed to a number of natural pathogens which are continually re-emerging from time-to-time by shifting from other host species where they have already established a complete reservoir of different variants. Such a switch over has often led to some of the most devastating epidemics recorded, including Human Immunodeficieny Virus/Acquired Immunodeficiency Syndrome (HIV/AIDS) in human communities.[1] This has even been argued that many microbial diseases affecting mankind (.like measles, tuberculosis, influenza and smallpox) emerged through pathogens jumping from domestic animals to humans on an evolutionary scale. A similar phenomenon also led to some of the devastating epidemics of plant pathogens in crop species like potato late blight in the cultivated potato and near extinction of American chestnut trees by chestnut blight.[2]

The etymology of the word pandemic can be traced to a Greek word *pandemos ‘pertaining to all people’ from pan -* ‘all’ plus *demos* meaning ‘people’. It could also be referred to as a widespread epidemic of contagious disease affecting whole of a country or one or more continents at the same time.[3] Human pandemics is a situation in which a local pathogen with epidemic potential gains and mutagenic ability affects humans on a national, regional or global scale. Its management requires active participation of various emergency functionaries with harmonization of their actions to avoid associated panic.[4] China and India, being thickly populated zones of South East Asian regions, are maximally vulnerable to such situations. India has already faced avian flu epidemics in the states of Maharashtra, West Bengal, Tripura. The continued presence of the pathogen enhanced the possibility of reoccurrence of incidence in future.

### Influenza outbreaks- transformation towards human pandemic

The actual account of influenza in humans probably dates back to the 12^th^ century, however, possible pandemics are documented as far back as 1510, but the first one to be clearly recognized occurred in 1580.[5] Retrospective analysis of sera collected from individuals born as early as 1857 revealed that the virus emerged in different strains leading to various pandemics. The reliable epidemiological data is, however, available from 1889-1892 pandemic onwards. Though influenza virus was first isolated in 1933, majority of the present day lessons learnt are based on epidemiological findings from 1918-1919, 1957-1958 and 1968-1969 pandemics[5]. At least two of these pandemics of the 20th century were associated with the re-emergence of such viruses similar to those which were circulated in previous eras, a process referred to as antigenic recycling[6] [Table 1].

**Table 1:** Emergence and re-emergence of influenza virus in 20-21^st^ century

Period	Influenza virus A	Observed global phenomenon
≈1889- 1901	H2N2	Retrospective analysis of sera collected from individuals born as early as 1857; caused 1889–1892 pandemic. Mortality rate were 10%, 45% and 45% in Jan-Mar, 1890; Mar-Jun, 1891 and; Dec- Mar, 1892 respectively in London[6]
≈1900 to 1918	H3N8	Mild pandemic in 1900
≈1918- 1919	H1N1	1918-1919 Influenza Outbreak*. Mortality rate were 5%, 60% and 35% in July, 1918; Nov, 1918 and; Jan, 1919 respectively in Copenhagen[6]
1957-1958	H2N2	Antigenic recycling and replacement of A/H1N1. Mortality rate were 43%, 28% and 29% in Oct, 1957; April, 1960 and; Oc, 1962Apr, 1963 respectively in United States[6]
1968-1969	H3N2	Antigenic recycling and replacement of A/H2N2 Mortality rate were 15% and 85% in March, 1968 and; Jan, 1969 respectively in England and Wales[6]
1977	H1N1	Re-emergence of virus after 20 years of silence and also co-circulation of A/H3N2; Pandemic alerts in military barracks of USA control limited its spread preventing a pandemic by mass vaccination program
1997	H5N1	Novel pathogen emergence in the persons exposed to infected poultry in Hong Kong; no significant human transmission
1999	H9N2	Two children exhibit influenza like symptoms due to the causative agent similar to A/H5N1 and possessed avian origin ∼ indicators for occurrence of pandemic
1999-2007	H5N1	Re-emergence as avian epidemic in different countries and in India, six-to-seven states were severely affected during different time periods; however their spread was controlled by stringent measures
2009	H1N1*	Emergence of H1N1 virus by genetic reassortment of endemic strain of human, avian flu and swine flu caused a pandemic situation

* Severe global pandemic outbreak

There have been regular outbreaks of influenza since 1996 to 2006 attributing towards genetics of the virus. It is pertinent to share knowledge regarding the virus and its virulence.[7] Recent episodes of influenza virus (H5N1) in South East Asian countries and other parts of world led global attention towards the impending pandemics. It is important to understand that evolution of Avian Flu pathogen capable of affecting humans will be inherently unpredictable, even though emerging pathogens tend to share some common traits. In case of direct transmission through RNA viruses, it was anticipated that they were most likely to jump between host species.[8] However, the spread of H5N1 virus from human to human transmission has not been observed in India.[9–10]

## PANDEMIC PREPAREDNESS STRATEGY

In view of the urgency of issue, WHO has now invoked the pre-established global pandemic preparedness plans and strategies for effective management..[11–12] The strategy encompasses following key performance areas: (1) capabilities-based planning based on cooperative governance, (2) equipping to manage various types of risks, (3) training and education towards development oriented approach, (4) exercising and evaluation to enhance reflexes and; (5) identification and incorporation of lessons learned.[13] In the aftermath of 9/11 incidence and anthrax attacks, there is an increased possibility of bioterrorism incidences leading to pandemics. It calls for preparedness that will transcend across the boundaries to manage any myriad mass casualty incidences. The looming potential for an avian (bird) influenza pandemic or an intentional usage of virulent microorganisms against civilian population is, therefore, a very distinct possibility.[14]

Impact will range from severe human sufferings to disruption of complete social system. It will eventually leads to a situation of helplessness and hopelessness. Inadequate preparedness will seriously delay overall detection and treatment efforts. An effective cooperation with authorities involved in surveillance, contact tracing and isolation measures; optimal distribution of resources for the prophylactic and therapeutic measures are important mitigation strategies requiring strict enforcement.[15,16] These mitigation strategies need to be complemented by local level/house hold based interventions which are effective tools to control spread of lower transmissibility strains during initial phase of spread of flu. Though community awareness and its active participation might reduce the number of incidences towards development of severity a combination of household-based quarantine, isolation of cases outside the households and targeted use of vaccines and specific anti-viral therapies appear to be highly effective and feasible.[17–18]

The present review outlines the inherent potential of switch over of avian influenza epidemic into a human pandemic, H1N1 pandemic 2009; major attributes towards emergency pandemic preparedness, health care preparedness, role of non-health care stakeholders and simulation exercises as learning tools for effective management of pandemics.

## EMERGENCE OF FLU OUTBREAKS AS FUTURE PANDEMICS

Influenza is a highly contagious, acute respiratory disease that spreads rapidly and pervasively through a population with diverse susceptible reservoirs of influenza virus. A mutated virus strain to which people have no immunity can cause influenza pandemic once the virus gains efficient and sustained human-to-human transmission capability. Extremely high case-fatality ratio among confirmed cases with genetic sequencing of influenza A (H5N1) virus from human cases in Thailand and Vietnam has been reported from 2004 to 2006.[11] In addition, significant resistance to antiviral medication like amantadine and rimantadine enhanced the probability of occurrence of influenza pandemic.[19]

**Figure 1:**
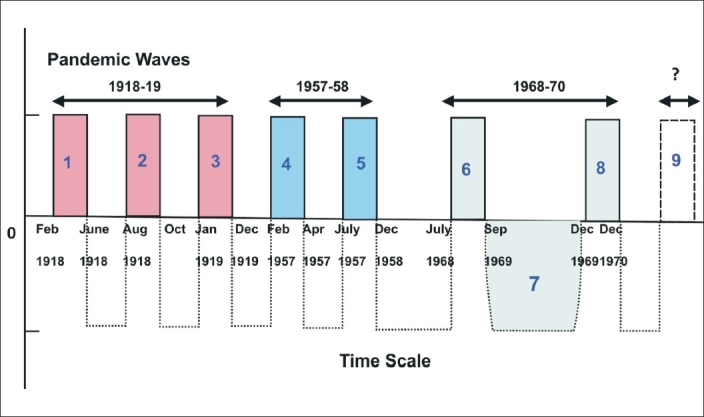
Global pandemic waves in 20^th^ century

A critical analysis of the occurrence of avian flu as shown in Table 1 reveals that pandemic waves occurred frequently in the 20^th^ century with an average interval of 11-39 years. Since the last pandemic occurred in 1968, 40 years have already elapsed, a series of incidences of avian epidemics with an aggressive avian influenza virus potential to spread to larger population indicating its inherent potential to sustain in the animal kingdom and ability to cause human pandemic [Figure 1].

### Avian influenza virus - low and highly pathogenic forms

Avian influenza virus uses protein marker hemagglutinin (H) to adhere and inject within a host and protein marker neuraminidase (N) is responsible for the virus' capacity to reproduce its progeny once it has infiltrated its host cell's DNA. These H and N proteins are used to classify different types of avian influenza viruses, and scientists have found that the H5 and H7 subtypes are the most likely to mutate and have potential to become deadly human pandemics.

Amongst them, H5N1 was found to have significantly high potential mutability.[19–20] Avian influenza exists in both low-pathogenic and high-pathogenic forms differing in its inherent ability to harm its host, and its capacity to spread between and among various host species. Low pathogenic avian influenza (LPAI) does not cause any illness in domestic poultry while an H5 or H7 LPAI strain posses significant potential to evolve into a highly pathogenic avian influenza (HPAI) strain.[19] HPAI is frequently fatal to domestic poultry and wild birds as it is easily transmissible between the susceptible species. The migration potential among different species across geographical boundaries led to the possibility of emergence of avian influenza virus as human influenza virus with a significant pandemic potential. Such jumps have already threatened species including lesser white-fronted goose, red-breasted goose, swan goose, oriental stork, Siberian crane and bar-headed goose. Recent reports from swine to human transmission have opened the pandemic potential of the virus.[11]

### Surveillance of human cases

Surveillance reports reveal more than 381 confirmed human cases with an average mortality of 60%.[21] The causative agent was reported to be H5N1 which belongs to category of HPAI viruses, progenitor genes of which are first detected in Eurasian landmass before 1966. The combinations among these progenitor genes generated at least 21 reassortants. Among these reassortants, H5N1-PR2 (responsible for Hong Kong outbreak) and H5N1-PR7 were found to be associated with confirmed human cases. H5N1-PR7 also contains a majority of the H5N1 viruses causing avian influenza outbreaks in birds, including the first wave of genotype Z, Qinghai-like and Fujian-like virus lineages.[22] These lineages are important towards pandemic preparedness as well as avian influenza prevention and control.[22]

### Effect of climatic and socio-economic factors

During inter-pandemic periods, in northern and southern temperate regions, the epidemiology of influenza is characterized by extremely low level transmission of influenza virus in summer months. It is generally followed by an annual upsurge in winter seasonal activity with clinically recognizable disease in the population for 8-12 weeks. On occasions, epidemics lasting four to six weeks, with an impact on both primary and secondary care were observed. In tropical and sub-tropical regions, although seasonal peaks of increased activity may be observed, the disease usually occurs year-round.[23]

In India, fresh outbreak of highly pathogenic avian influenza (HPAI) has been reported in North Dinajpur of West Bengal. It started on March 17, 2009 and affected 363 rural backyard poultry. Epidemiological investigation, culling within an area of three-km-radius and continued surveillance will be carried out in an area of 10-km-radius. There were reports of HPAI outbreaks in poultry in Darjeeling and Dakshin Dinajpur, Coochbehar districts of West Bengal and Ravongla district of Sikkim of India in January-February 2009. So far, no cases of human avian influenza have been reported from India. Last two years have seen a series of incidences in Maharashtra, West Bengal, Tripura and other parts of North-East. Due to the nonavailability of track and trace mechanisms, mass culling or killing happens when detected. In 2006 itself, Rs. 2200 crore have been lost. The porous borders, supply chain inefficiencies and inadequate infrastructure make it a regional level issue. The integrity of supply chain with adequate awareness strategy is an important mitigation strategy.[24]

On the other hand, sporadic occurrence of avian influenza outbreak is linked with annual wild bird migration and their interaction with local species, illegal poultry trade across the boundaries, local backyard poultry which comes frequently in contact with ducks and other species as well as porous international borders of developing countries. All these environmental and socio-economic factors enhance the level of spread of the virus across boundaries, calling for an effective global mitigation strategy.[22]

### Predicting future pandemic, impact

**Figure 2:**
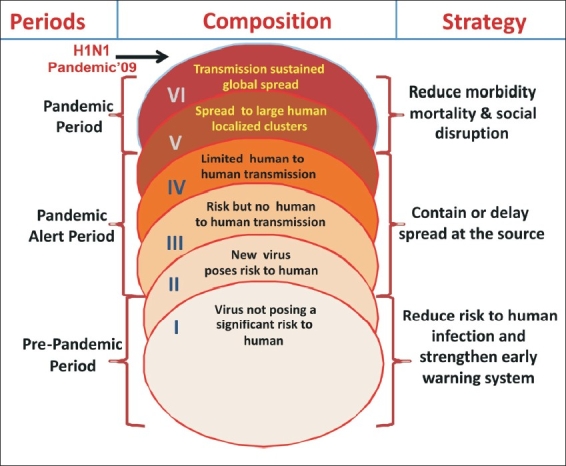
Composition of three pandemic periods (with six phases) suggested by WHO and corresponding mitigation strategy- H1N1 Pandemic'09 reached level VI

An extrapolation to an estimated impact of 1918-1919 Spanish influenza outbreak revealed that any future pandemic of similar nature might cause 180 million to 360 million deaths globally.[21] It will cause significant implications to the economy, national security, and the basic functioning of society leading to disruption of social structures. If the H5N1 virus acquires human-to-human transmissibility with its present fatality rate of more than 50%, the resulting pandemic would be akin to a global ‘tsunami’. The present volume, speed, and reach of travel has accelerated the spread of infectious diseases as observed in the case of Severe Acute Respiratory Syndrome (SARS), which spread to eight countries around the world in just a matter of weeks.[25] Such a deadly impact will not affect only the young population as in 1918.[26] The severe impact can spread to other sectors of the population including the old which is equally active outside the household boundaries and thereby equally vulnerable to be exposed.[25] In view of such a devastating potential, it is pertinent to develop national and regional plans complementary to the various pandemic periods illustrated by WHO [Figure 2]. As per the existing scenario, the world population is facing a novel mutagenic virus H1N1 at its pandemic proportions.

## TURNING TOWARDS H1N1 PANDEMIC - 2009

According to figures released by the US Center for Disease Control and Prevention (CDC) between 2005 and February 2009, a total of 12 cases of swine influenza were reported across 10 states in the US. However, as of 26 April 2009, the CDC reported 20 further confirmed cases in the US. It is speculated that the outbreak began in Mexico where, as of April 26, the Mexican Government reported 20 laboratory confirmed cases of swine influenza.[27] All these 20 laboratories confirmed cases had a novel viral strain; 68 persons died of severe pneumonia. The virus is being described as a new subtype of A/H1N1 not previously detected in swine or humans. Of the Mexican cases, 20 have been laboratory confirmed in Canada as A/H1N1, while 12 of those are genetically identical to the swine influenza A/H1N1 virus from California.[28] Mild influenza-like illness (ILI) was observed in all cases. An account of first 15 days of initial spread has recently been reported by us.[6]

In H1N1, the ‘H’ refers to the hemagglutinin protein, and the ‘N’ refers to the neuraminidase protein. It has a close similarity with H5N1 in terms of causing bursting of cytokines, fluid secretions into organs leading to state of breathlessness. The neuraminidase genetic segment of the virus provides it an ability to break out of infected cells. CDC refers the swine influenza A virus as A/Wisconsin/87/2005 H1N1.[29–30] There has been a continuous change in thinking and strategies based on changing level of risk with time. As reported in ‘Nature’ in May 2009, ‘the risk is not hyping the pandemic threat, but underplaying it’.[31] Within a month WHO declared the H1N1 outbreak at phase VI requiring immediate pandemic mitigation strategy.

H1N1 virus is a triple reassortant influenza virus with eight segments PB2 abd OA from North American Avian; HA, NP and NS from classic swine while NA and M from Eurasian Swine and; PB1 from human.[29] In comparison to H5N1, H1N1 can transmit between humans and cause mild disease. H5N1 doesn't contain molecular markers of pathogenicity and it may cause severe lung injury and death.[32] Reservoirs of H9N2 in children of Hong Kong and China; H6N1 in birds, H4N6 in ducks and other water birds and H5N1 in poultry birds, has been recently recognized with plausible pandemic potential[33] attributing to their sustenance in tropical climatic conditions of South East Asian countries.[33] H5N1 flu resulted in 261 fatalities from 424 human cases between 2003-2009.[34] Since H5N1 has an inherent ability to mutate, the proactive preparedness efforts were undertaken globally to effectively respond to any impending pandemic.[35] However, if H1N1 reassorts with H5N1 or any of the above-mentioned viruses, the subsequent virus may cause more severe pandemic.

### Analysis of spread of H1N1 pandemic during first 50 days

During the period of only 50 days (26 April 2009 to 15 June 2009), 76 countries officially reported 35928 cases of influenza A (H1N1) infection, including 163 deaths. If we compare the status 15 days prior to present day situation, the number of cases were half i.e. 17, 410 with 115 deaths. These figures show that the spread intensity is increasing but severity of the situation is still contained and requires focused attention for taking preparedness and mitigation measures at all levels.

The analysis of spread of H1N1 infection at an interval of 10 days from April 26 to June 15, 2009, revealed some important findings. The global increase in spread over this time period exhibited wave- like nature with lowering of spread half way (25-30 days i.e. May 26, 2009) and then peaking up. A similar pattern is observed in rate of increase of number of deaths with respect to increase in number of countries being affected [Figure 3a]. As suggested by the WHO, the overall severity of the influenza pandemic was found to be moderate based on the pandemic's impact on their health systems, and their social and economic functioning in 76 different countries.

**Figure 3a:**
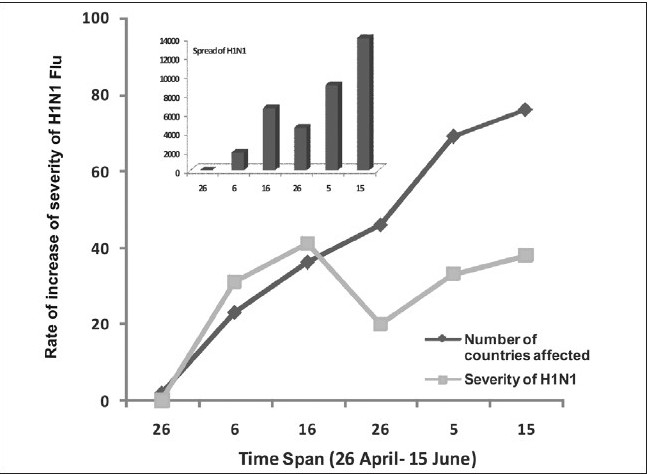
Rate of increase in severity of H1N1 infection (difference in number of deaths on n+10^th^ day- n^th^ day) globally with increasing number of countries being affected vs. time scale of 50 days (26^th^ April- 15^th^ June, 2009). Inset: rate of spread (difference in number of cases on n+10^th^ day- n^th^ day) at the same time scale (WHO update number- 12, 18, 30, 39, 44 and 49).

This assessment reflects that a) most people recover from infection without the need for hospitalization or medical care; b) overall, national levels of severe illness from influenza A(H1N1) appear similar to levels seen during local seasonal influenza periods, although high levels of disease have occurred in some local areas and institutions and; c) overall, hospitals and health care systems in most countries have been able to cope with the numbers of people seeking care, although some facilities and systems have been stressed in some localities. The underlying factors of any successful mitigation strategy are: (i) to identify a suspect based on clear cut ‘case definition’, (ii) an alert health care system and, (iii) an informed public to allay the panic.[36] It is also important to understand that due to panic associated with H1N1 virus; the seasonal influenza might be overlooked by government and general population in affected countries. Such ignorance may result in a large number of deaths.[37]

The ILI can cause a lowering of the immune system capability and a susceptibility to pneumonia. Although data on the spectrum of illness is not yet available for this novel influenza A (H1N1), clinicians expect complications to be similar to seasonal influenza: exacerbation of underlying chronic medical conditions, upper respiratory tract disease (sinusitis, otitis media, croup) lower respiratory tract disease (pneumonia, bronchiolitis, status asthmaticus), cardiac (myocarditis, pericarditis), musculoskeletal (myositis, rhabdomyolysis), neurologic (acute and post-infectious encephalopathy, encephalitis, febrile seizures, status epilepticus), toxic shock syndrome, and secondary bacterial pneumonia with or without sepsis. Since human cases have been reported with geographical spread of multiple community outbreaks and the unusual age groups affected (young adults); these events are of high concern. It is containable as it is susceptible to oseltamivir and zanamivir that can help to control the infection and help to prevent spread.

### Analysis of severity of H1N1 pandemic during first 50 days

The analysis of severity determined in terms of deaths occurred globally, are restricted to 163. The comparative analysis of major proportions of death occurred in Mexico (108) greater than US (45) greater than Canada (04) greater than Chile (02) with one death each in Columbia, Costa Ricia, Dominican Republic and Guatemala till June 15, 2009. Severity refers to number of deaths/number of confirmed cases and the comparative analysis of four major countries which have number of confirmed cases from the initial stage is illustrated in Figure 3b. The level of severity is also compared with global severity.

**Figure 3b:**
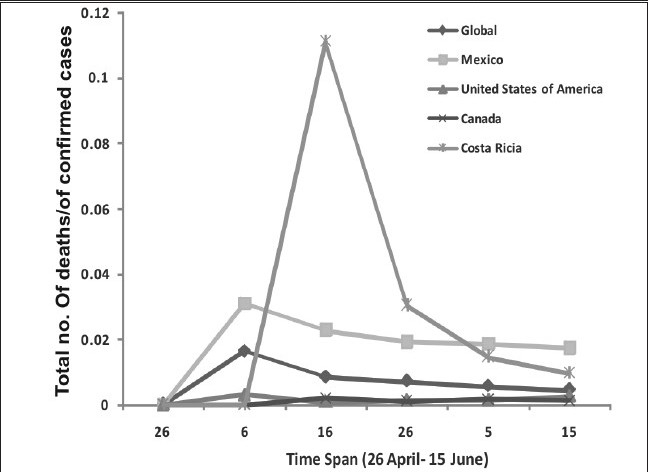
Countrywide differences in number of deaths / number of confirmed cases of four major countries with maximal number of deaths with respect to global severity index vs. time scale of 50 days (26^th^ April- 15^th^ June, 2009) (WHO update number- 12, 18, 30, 39, 44 and 49).

This analysis also exhibits the level of preparedness efforts which was found to be maximal in Costa Ricia followed by the United States, Canada and least in Mexico. However, the data based analysis doesn't give an accurate picture as there are number of confounding factors which control the overall situation. It includes population of the country, number of health care facilities, genetic profile of the community, living style and other socio-environmental factors etc.

US agencies have defined severity on a scale of 1-5 based on the range of case fatality rates from les than or equal to 0.1% (for severity - 1) to greater than or equal to two per cent (for severity - 5). Another variable is the time lag in which an infected individual start infecting others. It is probably between three to five days, but closer to three days.[38] The global mitigation approach reflects major concerns related to current patterns of serious cases and deaths that are occurring primarily among young persons, including the previously healthy and those with pre-existing medical conditions or pregnancy. Large outbreaks of disease have not yet been reported in many countries, and the full clinical spectrum of disease is not yet known.

### Predictive assessment of H1N1 spread in India during next 50 days

Mexico and US are the primary countries affected by the H1N1 infections, the ratio of cases on each 10^th^ day with respect to the nth day (n=0, 10, 20, 30, 40) was assessed and illustrated in Figure 3c. Analysis revealed a significant increase in number of cases in first 10-20 days followed by steady decrease in rate. Based on the average of ratios of Mexico, US and global cases; the expected rise in number of cases in India from existing confirmed cases of 16 (as on June 15, 2009, in accordance to WHO update 49) an anticipated rise to more than 300 in next days was expected. Though, due to lack of medical preparedness at state level, the number roses even to more than 1000 cases in this stipulated period with 0.01% case fatality rate till August 15, 2009 It is expected to rise further in an exponential rate and at significantly different rates in various states of India. In Mexico, the case fatality rate was estimated to be around 0.4%[39] while in countries other than Mexico, an estimate revealed approximately 18 deaths in 12,381 cases, confirmed for causative agent i.e. H1N1 virus (as per WHO update no. 42). However, the severity of pandemics can change over time and differ by location or population. Close monitoring of the disease and timely and regular sharing of information is critical during the pandemic period is essential to determine future severity assessments, if needed. Future severity assessments would reflect one or a combination of the following factors including a) changes in the virus, b) underlying vulnerabilities, or c) limitations in health system capacities.

**Figure 3c:**
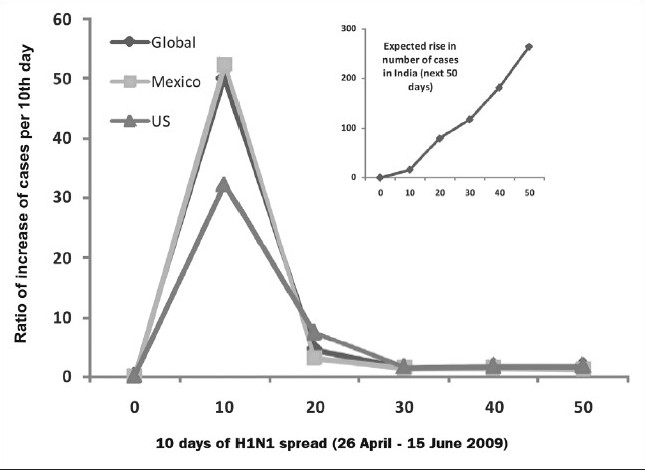
Ratio of increase in number of confirmed cases of H1N1 infection (number of cases on n+10^th^ day / n^th^ day) at global, Mexico and US at a time scale of 50 days (26^th^ April- 15^th^ June, 2009). Inset: expected rise in number of cases in India in next 50 days based on the average ratio of increase of global, US and Mexico spread in last 50 days with starting point of officially reported 16 confirmed cases in India (WHO update- 49).

### Approach of WHO towards H1N1 pandemic

After an emergency meeting, the WHO has finally declared a global flu pandemic, a first in 40 years after the 1968 Hong Kong flu that killed approximately one million people. It raised the global pandemic flu alert to level six[26] (the highest level of spread) on June 11, 2009 from its previous level declared on April 29, 2009. Pandemic alert VI level indicated that now member states (countries) should take necessary travel restrictions, if required, as well as need to take all possible actions to contain the situation to limit its severity. The major step is to strengthen and support health systems in countries with fewer resources. Health systems need to be able to prevent, detect, treat and mitigate cases of illness associated with this virus. WHO is also stocking medicines (such as anti-viral and antibiotics) so that it becomes more accessible and affordable for developing countries.

The important underlying fact is that patients are responding effectively to tamiflu (antiviral drug) which has restricted the deaths to 163. However, it is known that virus keeps on mutating, and a new strain might develop resistance to present therapy. Further, winter upsurge might provide it an opportunity to damage more severely. It is essential to enhance the level of preparedness and to develop adequate contingency plans to manage such critical situation. This calls for immediate preparedness efforts towards development of adequate vaccines for some specific traits and multi-sectoral global mitigation approach to contain the plausible worst case scenario. Till now, the antiviral drugs and vaccines have targeted three influenza proteins i.e., hemagglutinin, neuraminidase and matrix 2 ion channel protein, though, it is important to explore new targets like polymerase complex activity, interferon antagonist activity and virus assembly etc.[40]

## EXISTING FRAMEWORK FOR PANDEMIC PREPAREDNESS IN INDIA

Enactment of Disaster Management Act, 2005 led to establishment of a national, state and district level disaster management (DM) authorities. These authorities work in all phases of DM cycle i.e. prevention, preparedness, mitigation, response, recovery and rehabilitation. They work in coordination with nodal ministries/ departments and other stakeholders at all levels. As a member state for compliance with international health regulations (2005); India has developed core competency to detect, respond, rapidly to public health events to report within 24 hours to WHO.[21,41]

### Stakeholders for management of pandemic

National Disaster Management Authority (NDMA) is a national apex body, assisted by the NEC (National Executive committee), and acts as a national level coordinator and facilitator for all the concerned activities on disaster at national and regional levels. Ministry of Health and Family Welfare (MoHandFW) has the responsibility of providing guidance in surveillance, outbreak investigation, deployment of rapid response teams (RRTs), associated manpower and logistic support for effective management of worst case scenarios. National Institute of Communicable Disease (NICD) is the nodal agency for investigating outbreak and also provides teaching /training, research-based interventions and adequate laboratory support in participatory mode with Indian Council of Medical Research (ICMR). State Disaster Management Authority (SDMA) needs to develop plans based on national guidelines on management of biological disasters which are already in place. It is assisted by SEC (State Executive committee) and provides adequate guidance and support to integrated disease surveillance program (IDSP).

District Disaster Management Authority (DDMA) has been designated with the responsibility of planning and execution by coordinating with both health and non-health stakeholders/ service providers. Primary health centers are the peripheral units that provide preventive health care and it is necessary to have one PHC per 30,000 individuals. Anganwadi workers/auxiliary nurse midwife (ANM) are the available manpower requiring training and awareness. PHC looks after health related matters at local level with the help of sanitation committee and municipal corporation. At all these levels, Department of Animal Husbandry has its respective units at different levels to contain the virus with in poultry sector itself. In addition, various service providers in health and non health sector are also required to be in loop for comprehensive management. ICMR initiated the process of development of vaccine within India to become self-reliant. ICMR has short listed three companies which will produce these vaccines in different parts of the country. The complete process will take nearly four to six months.[42] The Union Government of India have installed thermal scanners at all major entry points and make screening mandatory for all incoming passengers from the flu-affected countries.[43]. Keeping the gravity of situation under consideration by August 15 2009, the central government has developed mechanism to provide significant quantities of Tamiflu to all the states, different cultural activities are being monitored, schools in highly affected areas are closed for a certain period of time, private hospital resources are also pooled and newer diagnostic facilities are being developed across the country to manage the situation effectively.

*IDSP:* This program *w*as launched in 2004 to keep a watch on disease outbreak and also provide necessary data to monitor the progress of ongoing disease control programs. IDSP has the responsibility of disease surveillance at the state and district levels, improvement in laboratory support, strengthening data quality and analyzing linkages among them. Surveillance initiates at block level 0-3 km daily and 3-10 km bi weekly. Surveillance workers should have PPE and chemoprophylaxis along with good facility for collection and transportation of human sample. IDSP is an effective preventive tool in place to contain the emergency in restricted regions. National surveillance program for communicable diseases has been launched to strengthen the disease surveillance system so that early warning signals are recognized and appropriate timely follow-up action is initiated. The main objective of the program is capacity building at district and state levels. Ministry of Agriculture (MoA) has one BSL-4 laboratory at the High Security Animal Disease Laboratory (HSADL) at Bhopal which received all samples of avian flu. Another laboratory is at National Institute of Virology (NIV), Pune, working in the area of detection.[44]

### HEALTH CARE

Health care preparedness includes a) preventive measures to limit the spread of avian flu from poultry sector to humans by capacity development of veterinary heath care sector; b) hospital preparedness; c) critical infrastructure development; d) national pharmaceutical stockpile program; e) national and state level health care planning as a part of institutionalized disaster management framework and; f) regional and global level health preparedness with WHO as a focal point of convergence of resources and sharing of medical logistics to contain its spread at the epicenter of such pandemics.[36]

National Institute of Communicable Diseases, Delhi has issued a special issue of their publication ‘CD Alert’ on human swine influenza with relevant information usable by health professionals and community.[45] It is essential to follow all infection prevention / control measures during pre- and hospital care along with specialized instructions for similar stringent measures in isolation facility.[46]

### Hospital preparedness

The hospital preparedness, being a primary issue, should include: a) plotting of worst case scenarios of pandemics vs. available resources; b) identification of all possible solutions like enhancement in number of beds, utilization of multiple ventilators, emergency stockpiles, networking of hospitals to pool the local resources, advanced equipment (modes to quickly procure them), mechanisms to rapidly create temporary isolation facilities, systems to restrict access to exposed health care workers[47] and plans to involve specialists to screen and identify cases at an early stage with continuous monitoring to ensure adherence to optimal infection-control practices; c) recognition of level of effective care and adjusted emergency preparedness protocols; d) specialized contingency planning for worst case scenarios where hospital itself gets contaminated due to spread of biological agents.[48] The preparedness needs to include rural, urban hospitals coupled with both specified training, and table top exercises to identify/analyze weak spots, development of regional teamwork, and rapid response teams to respond to any such emergency.[49]

### Mass vaccination program

Speed and acceleration of spread of pandemics can only be restricted by mass vaccination program. However, the success of any such program depends upon the critical infrastructure in place to identify the causative organism, its characterization, mapping of genome to identify specific traits which can be targeted to develop an adequate vaccine. Such vaccination leads to the formation of memory B cells generating antibodies against the virus which will destroy the virus directly.[50] The limitation is that development of any specific vaccine requires time and delay which will continuously add up millions of deaths. It is important to understand that the existing variants of influenza virus are being tested on the common traits which, predictably, may not mutate while attaining potential to affect human population. The vaccination program using these vaccines might be effective or may not work at all. One common misconception about flu vaccination is that it prevents infection with flu entirely. This is not so. Flu infection occurs even if one has been successfully vaccinated against that strain of flu. The patients may have some mild common cold symptoms or may not have any symptoms at all.[51] However, if the mutation is highly lethal and existing vaccines like sanofi pasture H5N1 avian flu vaccine and others do not work against the offspring virus, such pandemic will certainly lead to mass casualty events across the globe.

Developed countries have already developed effective vaccines for their entire population but it may constitute only five per cent of the total global population.[52] Recent studies showed that young healthy adults become immune with a reduced (half) dose of killed flu protein if it is given combined with an adjuvant. Such technology will eventually be an effective tool as H5N1 candidate adjuvant vaccine for cross-protection against lethal H5N1 challenge in ferrets.[53] As observed in previous pandemic waves [Figure 1], the first wave might affect millions in one go followed by a latency period which should be utilized effectively to produce a viable vaccine against newly evolved virus. Table 2 illustrated some efforts in development of vaccines in last five years.[54–56]

**Table 2:** Some important vaccines developed for medical management of pandemics[48–50]

Year	Industry	Vaccine type	Brand name	Salient feature(s)
2004	Sanofi Pasteur	Inactivated vaccine	-	It is an inactivated vaccine made from an H5N1 virus isolated in southeast Asia in 2004. Vaccine is produced from virus grown in fertile hen eggs and inactivated by either formaldehyde or beta – propiolctone. It is given by intramuscular injection.
2006	MedImmune	Live attenuated influenza vaccine (LAIV)	MedImmue FluMist R influenza vaccine	This vaccine is based on combination of modified protein derived from virulent H5N1 flu virus with protein from an attenuated flu strain. LAIV has been license by FDA in 2003. It contain live but attenuated (weakened) influenza virus. It is sprayed into nostril instead of injected into muscle.
2008	-	Cell culture vaccine	-	Vaccine is created by using cell of monkeys instead of chicken's egg which reduces the time of development of vaccine.
2009	-	Recombinant vaccine	-	Use of Human Monoclonal antibodies that neutralize different strain of influenza A virus. Subunit influenza vaccines have been prepared from recombinant haemagglutin and neuraminidase protein expressed in insect cells by baculoviruses.
2009	NOVAVAX	DNA vaccinevirus like particle (VLP) vaccine candidate	-	Provide broad protection against strains of Avian Infiuenza, mainly 3 strains are used A/Qinghai(clade2.2),aaA. It present promising approach to vaccination, evoking full range of immune response. DNA vaccines with constructs encoding the nucleoprotein(NP), haemagglutin, neuraminidase, matrix protein 1(M1) and non structural protein 1 of influenza virus.
2009	Delsite Biotechnologies	Live attenuated vaccine	Gelvac TM Nasal powder	It is preservative and adjuvant free and allows needle free administration and it reduce the cost of stockpiling of strategic vaccine.

The clinical symptoms of the disease appear to be mild, though complications may occur if there is an underlying lung or cardiac disease, diabetes or those on immunosuppressive therapies.[57] Throat or nose swabs are suitable for detection which are confirmed by specific RT-PCR Assay that differentiates it from seasonal flu virus followed by isolation / identification of H1N1 virus and then detection of a fourfold rise of neutralization or HA1 antibodies to H1N1.[58]

### Anti-viral drugs

Predictions revealed that 25-50% of the population will become sick during first wave itself. The number of patients during the first wave of pandemic depends upon the clinical response to anti-viral or anti-flu antibiotics.

### Oseltamivir therapy

Oseltamivir is an orally active antiviral drug that is used in the treatment and prophylaxis of both influenza A and B. It is currently marketed under the trade name tamiflu. It is a neuraminidase inhibitor and prevents new viral particle from being released by infected cells. If treated successfully, it needs no further vaccination. The WHO has recommended that every country establish a stockpile of enough drugs to treat 20% of its citizens to manage any possible avian influenza pandemic. It works effectively if it is given early within the first 48 hours of the illness with a dosage of one tablet for five days. However, it has some common adverse drug reactions (ADRs) of such therapy like nausea, vomiting, diarrhoea, abdominal pain and headache. In 2007, Japanese investigators detected neuraminidase-resistant Influenza B virus strains in individuals who had not been treated with these drugs. The prevalence was 1.7%. It indicates that it might not work if the mutation favours such resistance.[59] The indiscriminate use of antiviral drugs may lead to drug resistance. One of the strategies to prevent drug resistance may be usage of both therapies i.e. zanamivir and oseltamivir.[60]

### Zanamivir therapy

Another anti-influenza antibiotic that might be effective against H5N1 avian flu is Relenza^®^ or zanamivir, though has not been completely established yet. Zanamivir is a transition state analogue inhibitor delivered through an inhaler used in the treatment and prophylaxis of both influenza A and B. If given within six to 12 hours, it is ideal for treatment. It may cause bronchospasm so it should be administered with apparent safety in patients with asthma or chronic obstructive pulmonary diseases. It is specific and has not been known to cause toxic effects as it does not spread around through the body's systemic circulation. It also shows no signs of viral resistance yet.

### Limitation of health care interventions

Avian flu has been found to be resistant to the other older anti-influenza drugs like amantadine. It is important to understand that medical preparedness has the limitation of failure of prediction of understanding virus. The important factor to be considered is that even during first wave, the hospital health care facilities will be exhausted, lot of health care workers may themselves get affected or died, hospital will quickly run off supplies and medical logistics due to shortage of other laboratories and absenteeism in pharmaceutical industries and other industries.[61] It is important to develop flu survival kit including various household items like ibuprofen, acetaminophen, table sugar, and table salt, thermometer, an automatic blood pressure and pulse monitor required to manage it effectively during primary attack. The national stockpile program is required to be in place for development of stockpiles at strategic location across the country. However, any such program will remain ineffective unless and until essential services other than health sector are not in place and secured.

### Medical management of H1N1 - Some recent initiatives at national level

National Disaster Management Authority has been assigned with the responsibility of facilitation of all the activities / mitigation efforts undertaken by nodal Health Ministry and other departments/ ministries concerned. Accordingly, NDMA/nodal ministry have issued a number of advisory documentation for undertaking necessary preparedness measures. Earmarking of hospitals, stockpiled drugs at different locations, training of doctors and paramedics, mechanism to provide care to care providers, special teams to manage vulnerable community, development of Rapid Response Teams (with minimum of one medical and paramedical staff), development of isolation wards, mechanism to transport the affected victims to the nearest earmarked hospital sites.

## PREPAREDNESS OF NON-HEALTH SECTOR

Lessons learnt from previous preparedness efforts applicable towards existing preparedness efforts include: a) testing of validity of models developed using different indicators as contact evidences; b) research and contemporary preparedness should go simultaneously with a cautious balance;[62] c) operational responsibilities of health and non-health administrators; d) balanced approach towards overstatement of objectives and misrepresentation of overall risk contained; e) strengthening of local capacity for effective implementation; f) strategic communication; g) program review and; h) continuous improvement of existing level of preparedness efforts.[63]

### Mitigation strategy in pandemic alert period

**Figure 4:**
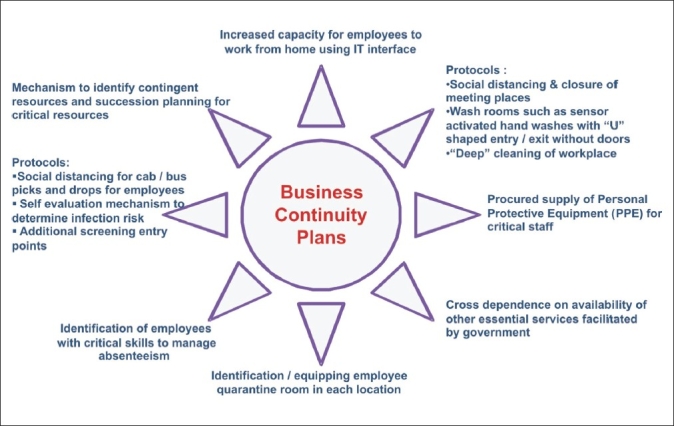
Components of suggested business continuity plans for pandemic preparedness

H1N1 outbreak has now been declared as pandemic [Figure 2]. It is essential on the part of global aviation community to limit/contain the spread of virus across the world. Each member country's level aviation sector needs to define a contact focal point for regional preparedness and its interlinking with national level health sector. Adoption of sensitization programs for all passengers about the various risks to which they may be exposed in different parts of world by different modes of communication and transportation. Various emergency functionaries as well as essential service providers need to be maintained continuously as their active role is pertinent for effective management of pandemics[21,44] [Table 2]. Emergency preparedness efforts need to include business continuity planning by various organizations, service providers, corporate groups, financial institutions and industries.[24] The components of business continuity plans[64] are illustrated in Figure 4. All these plans need to be tested in simulated and controlled environment.

### Essential service provisions of non-health sector - recent initiatives in H1N1 management

NDMA has taken up this task proactively in 2008 and released a document stating the role of non-health sector in pandemic preparedness. For regional level preparedness of such sectors (across the boundaries in South East Asian Region), utilizing South Asian Association for Regional Cooperation (SAARC) should take necessary steps in such directions. Based on the analysis of present status, prior to switch over to VI level, NDMA has issued specific directions to various ministries specifying their roles in such incidences and mechanism to implement all these activities. Table 3 illustrates role of various non health service providers in managing this pandemic situation.

**Table 3:** Role of non-health service providers

Non-health sectors	Service providers	Composition and roles
Financial services	Banks, financial institutions, stock exchange etc	Maintenance of bare minimum financial services with inherent mechanism to ease control
Utilities, personal service	Electricity, water, food, telecoms, postal service, retailing (catering for the needs of the most vulnerable)	Contingency plans to provide services in integrated manner
Transportation-logistics, business, leisure: supply system	Air, sea, rail, ports, pilgrimages, sports and other events, tourism	Key control for all services, special plans to provide assistance in managing absenteeism
Government, security and armed forces	Public service, rule of law, judiciary and correction, private security, respect for rights	Provide framework, ease on regulations, stringent control by applying emergency laws in place
Information and management	Transparency, strategic communication, broadcast and print, internet	Economic backbone and strategic business continuity planning is necessary
Environment and hygiene	Cleaning, maintenance, refuse management, wildlife	Needs to place to prevent spread of infection
Food and livestock production	Growing, processing, marketing and distribution of animal meat for human consumption	Basic need requires replenishment through stores stocks, if necessary

## SIMULATION EXERCISES: EFFECTIVE TOOL FOR PANDEMIC PREPAREDNESS

In view of the magnanimity of the pandemic situation and involvement of various agencies, the preparedness, measures also call for periodic brain storming of various stakeholders cutting across the physical boundaries.[21,44] Simulation exercises and table-top discussions on relative issues of pandemic scenarios are pertinent tools of awareness to manage complex queries. Such exercises should have focused objectives with a defined scope and need to simulate a worst case scenario. Tasks to be performed by different participants or emergency functionaries, assessment of their critical needs and role for various facilities are tested in controlled environment. Teams are monitored; flow of action controlled and as observers of exercise, neutral weak areas need to be identified.[65] In addition, the experiential exercise is an effective, inexpensive, and easily able tool for promoting multiple competencies in mass health emergency preparedness for a variety of health care students including medical, veterinary, public health, and nursing students and to test operational capacity and response readiness of various responders.

There is an urgent need for similar exercises in India, at regular intervals, organized by NICD in cooperation with Ministry of Defence and NDMA; sharing recommendations in public domain.

## CONCLUSIONS

Influenza A pandemic is no longer hype or horror but a reality spreading at a very dangerous speed. It is the responsibility of every stakeholder/essential service provider, policymaker, decision maker to act proactively to prevent/prepare for any such emergency situation. India has proactively launched Integrated Disease Surveillance Programme (IDSP) as part of national health programs. NDMA issued national guidelines for management of biological disasters in July, 2008 as mandated by DM Act, 2005. Guidelines for preparedness/mitigation of pandemics especially by all non-health care providers which need to be integrated with pandemic preparedness health plan were developed by Department of Health and Family Welfare as per WHO guidelines. They include public private partnership models to maintain business continuity of essential sectors and special provisions for different vulnerable groups to be included as a part of overall plan.

Media management is an effective tool to alleviate stress or panic among the civilian population and utilization of this sector in a disciplined and effective manner is a complex task required to be proactively amalgamated in the overall preparedness efforts. Besides protecting their own citizens, various countries should work in unison towards global mitigation strategy.[66] Adequate preparedness of every essential service provider to sustain their services during pandemics is important. Practising the pandemic medical management plans through simulation-based exercises enhance the level of reflexes and augment the operational readiness. In addition to health sector plans, the execution of business continuity and contingency plan with optimal use of man and material resources is critical to develop disaster resilience. Contingency planning also needs to be in place;[67] if H1N1 again reassorts (by reentering into pigs population) either with seasonal virus or H3N2 virus in pigs, it could lead to changes in its replication and virulence as well as probability of anti viral drug resistance.[57]

In all such cases, the present mitigation strategy might not be effective. It is apparent to promote a global collaborative approach from health care experts, government and media, to control and curb the spread of infection worldwide.[68] In conclusion, it is the right time to execute the plans and ensure optimal utilization of all resources to respond effectively to impending waves of present day emergency of H1N1 pandemic.
